# Factors associated with cessation of smoking in health professionals: a scoping review

**DOI:** 10.1080/16549716.2023.2216068

**Published:** 2023-05-31

**Authors:** Amanda Evenhuis, Stefano Occhipinti, Liz Jones, Darren Wishart

**Affiliations:** a School of Applied Psychology, Griffith University, Brisbane, Australia; b International Research Centre for the Advancement of Health Communication, Department of English and Communication, The Hong Kong Polytechnic University, Kowloon, Hong Kong; c Department of Psychology, Monash University Malaysia, Subang Jaya, Malaysia

**Keywords:** Tobacco use, health professionals, tobacco control, public health, smoking cessation

## Abstract

**Background:**

Offering cessation support to health professionals who smoke to ensure optimal implementation of cessation support for patients is a key recommendation of the WHO Framework Convention on Tobacco Control Article 14 guidelines. However, direct efforts to support this population to quit are limited. Although numerous articles on the topic of tobacco use among health professionals have been published, the factors associated with their own cessation have not been systematically synthesised.

**Objective:**

We sought to synthesise existing literature on the predictors and processes informing attitudes and beliefs of smoking health professionals’ own cessation.

**Methods:**

A five-step methodological framework for scoping reviews was followed. We conducted a systematic search of EMBASE, PubMed, Web of Science, and PsycINFO databases, as well as Google Scholar for relevant articles. Titles, abstracts, and full texts were screened against predefined criteria: research published between 1990 and 2021, in English-language peer-reviewed journals; participants included doctors, nurses, medical, and student nurses who smoke.

**Results:**

The initial search yielded 120, 883 articles, with 27 selected for synthesis. Prevalence estimates and predictors of smoking behaviour have remained the primary focus of smoking health professional research. Few studies explicitly examined the relevant predictors of quit attempts and quit attempt success. There is evidence that age and work environment factors predict quit attempt success in some health professional groups. There is also some evidence of tobacco smoking stigma experiences among nurses and nursing students who smoke.

**Conclusion:**

Although cessation support is desperately needed for health professionals who smoke, the evidence for factors predicting quit success remains limited. To better guide future research, first, more theoretical work is required to identify the relevant predictors. Second, these should be tested using prospective research designs that take a multi-focal perspective to clarify the targets for change.

## Introduction

Owing to the direct impact of tobacco use on health, smoking cessation remains a key pillar of the global health agenda [1,2]. Reducing the number of current tobacco users by way of cessation support is a key policy focus of the World Health Organization [3–5]. Health professionals occupy a crucial role in cessation interventions [5–7], given their involvement is both efficacious and cost effective [8–10]. However, a small but non-trivial proportion of health professionals continue to use tobacco [11,12], posing a threat to the optimal implementation of cessation support [7], because doctors and nurses who smoke conventional tobacco are 17% and 13% less likely, respectively, to deliver cessation advice to patients [13,14]. More recently, studies have indicated that medical and nursing students who currently smoke conventional tobacco are also more likely to use electronic cigarettes (e-cigarettes) and heated tobacco products [15–17], and less likely to reduce their conventional smoking [17]. There have been similar findings among French military nurses [18]. Yet, direct efforts to support smoking health professionals to quit are not only limited [19–22], the effectiveness of these interventions is lacking [23]. Notably, the field lacks both an evidence base and clear guidance to inform targeted, relevant behavioural cessation interventions to support the unique needs of smoking health professionals. The lack of relevant cessation interventions to support this population may be driven by the incomplete narrative provided in reviews of tobacco use among health professionals to date.

Several reviews have been conducted on the topic of tobacco use among health professionals, however, these have focused primarily on prevalence estimates [12,22,24–28]. Although reviews of prevalence estimates are vital to inform policy makers about the scale of the problem and the required mobilisation of resources, further studies are required that provide a coherent base of theory and behavioural evidence to inform interventions sensitive to the personal and professional characteristics of this group. This includes context-specific factors and dynamics that may impact public health behavioural change interventions with health professionals across cultures [29,30]. One particular social issue that warrants consideration is the widely accepted unintended consequence associated with tobacco control strategies, the stigmatisation of smokers [31,32]. Two systematic reviews propose that the stigmatisation of smokers may in and of itself undermine cessation efforts among residual smokers in the general population [33,34]. The question remains as to whether this stigmatisation that is occurring within the broader context of tobacco control efforts influences the cessation of smoking health professionals. An understanding of smoking attitudes and beliefs from a psychological perspective, and other predictors of cessation of smoking in health professionals are required for an improved understanding of cessation success [35,36].

A scoping review was considered the most appropriate method, as it seeks to convey the breadth and depth of available research on a topic, identify research gaps, and make recommendations to inform policy and future research [37]. Accordingly, the aims of the present scoping review were: (a) to map the existing literature published since 1990 to identify and assess the factors that may impact cessation for health professionals who smoke; (b) to identify knowledge gaps; and (c) to include recommendations on how to move research on this distinct population forward.

## Methods

The scoping review followed the original methodological framework outlined by Arksey and O’Malley [38] and refined by others [39]. The method and five stages are described below. To ensure rigour and guide reporting, the PRISMA Extension for Scoping Reviews [40] was followed (see PRISMA-ScR checklist Appendix A).

### Identifying the research question

The following research question guided the scoping review: What are the factors impacting the cessation of smoking in health professionals?

### Identifying relevant studies

We searched PubMed, EMBASE, PsycInfo, Web of Science, and Google Scholar. The search terms covered the population and outcomes relevant to the research question, and included overlapping terms to ensure the widest possible scope of studies were identified in the search across the electronic databases (see Table 1). A copy of the PsycInfo search strategy is included (see Appendix B). The second step involved reviewing the reference list of studies identified in the electronic database search to identify any additional studies. Searches were conducted between June and July 2019. An updated search was conducted in January 2022.

**Table 1.** Search terms used.

Population	Nurse* OR registered nurse OR primary care nurse* OR nursing staff OR nursing profession OR physician* OR primary care physician* OR doctor OR healthcare worker* OR healthcare professional* OR health care professional* OR health professional* OR health service* OR medical profession* OR hospital OR hospital sites OR teaching hospitals OR medical student* OR nursing student*
AND Outcomes	Smok* OR smoking behaviour OR smoking behaviour OR smoking habits OR tobacco OR nicotine OR cigarette*
AND	Tobacco control OR smoking cessation OR smoke free hospitals OR tobacco denormalization OR tobacco denormalisation OR smoking denormalization OR smoking denormalisation OR moralisation of smoking OR moralization of smoking

We identified studies published between 1990 and 2021, with aims targeting predictors regarding tobacco use and cessation among doctors and nurses who smoke. Medical and nursing students were also included in the population, given the concern their attitudes may persist until they practice, potentially impacting their cessation responsibilities in the future [11,41]. The search year of 1990 onwards was selected for the following reasons: (1) it corresponds with the intensification of tobacco control initiatives in the general population [42,43]; (2) the adoption of the *Treating Tobacco Use and Dependence Guideline* in 1996 and subsequent updates [7,44,45], the adoption of the WHO FCTC in 2003 and entered into force in 2005 [3], the *Code of Practice on Tobacco Control for Health Professional Organizations* in 2005 [6], and WHO’s policy focus, *Offer help to quit tobacco use* [5]. The database search results were exported into EndNote for screening.

### Study selection

**Figure 1.**
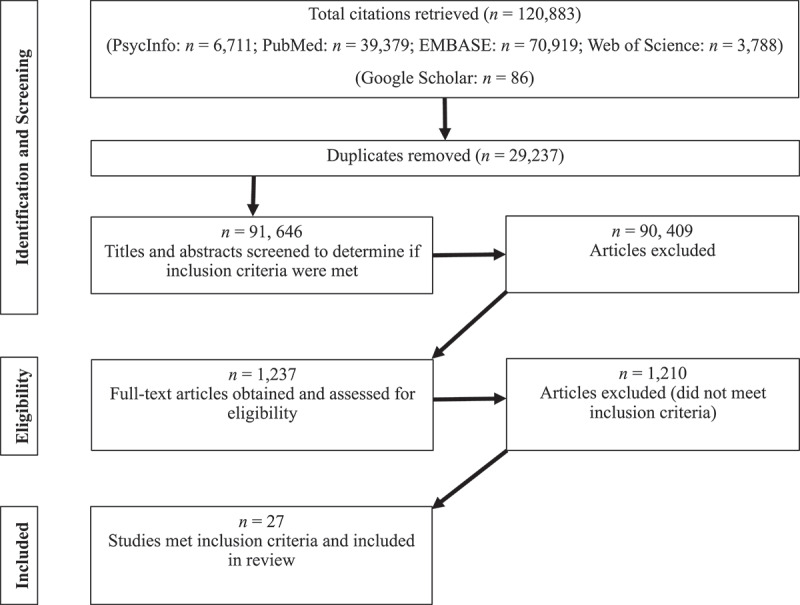
Literature review flowchart.

A PRISMA-ScR flow chart (see Figure 1) documents the search and study selection process. The search strategies generated an initial pool of 120, 883 articles. Using the EndNote database, 29, 237 duplicates were identified and removed. The titles and abstracts of the 91,646 articles were reviewed, where 90,409 articles were removed. The remaining 1,237 articles were read in full, resulting in the removal of an additional 1,210 articles.

The following pre-defined inclusion criteria were applied: (a) research published between 1990 and 2021; (b) published in English; (c) participants included current or former smoking health professionals expected to be directly involved in the delivery of cessation support to patients, that is, medical doctors, nurses, dentists, medical, and nursing students. Participants generally considered allied health (e.g. midwives, community healthcare workers) but combined with health professionals were also included; (d) studies that reported analyses and findings of factors predicting either quit intention, quit attempts, or quit attempt success; and (e) reported data collected through quantitative, qualitative, or mixed methods.

The following pre-determined exclusion criteria were applied: (a) prevalence only studies; (b) reviews, abstracts, dissertations, books, commentaries, editorials, and letters; (c) KAP studies concerning other populations (i.e. non-smoking health professionals, general population, pregnant women, HIV-positive patients, mental healthcare consumers, incarcerated and homeless persons); (d) epidemiological studies where tobacco smoking was a risk factor; (e) post-operative complications associated with tobacco use; (f) health professionals’ attitudes towards general population tobacco use; (g) studies solely investigating use of other tobacco products (i.e. smokeless tobacco, electronic cigarettes, and waterpipes) and other smoking behaviours (i.e. medicinal or recreational cannabis, cocaine, and opium); and (h) surgical, wildfire, and housefire smoke complication studies.

Initially one author (AE) screened retrieved titles and abstracts for eligibility. The full text of potentially eligible articles was retrieved, and AE reviewed these to determine whether they met the inclusion criteria. Two authors (SO and LJ) double screened 10% of eligible articles, respectively. The identified studies then underwent a second stage of screening where each full-text article was read (AE) to determine if the full inclusion criteria were met. The reference lists of these articles were reviewed, however, no additional articles were identified for inclusion.

### Data charting

Data were extracted and tabulated into Microsoft Excel (2019). Two templates were generated for this stage. The first template involved charting the descriptive data for each study: authors, publication year, country, study design, setting, sampling method, sample size and makeup, materials used, response rates, smoker definition used, and method of identification of smoking status. Countries were aggregated according to the six WHO regions. Another template was developed to collect data on the review question. Data extraction was undertaken independently by AE and a research assistant, with conflicts resolved through discussion, and a final decision was agreed upon. A copy of the data extraction table is available upon request by contacting the corresponding author.

### Collating, summarising, and reporting results

The heterogeneity of study methodologies necessitated a qualitative rather than quantitative meta-analysis synthesis of research findings. This approach is distinct from reviews that undertake a thematic summary of qualitative research [46,47], and consistent with the approach specific to scoping reviews [39]. There was no formal quality assessment of studies undertaken in line with the nature of scoping reviews [38].

## Results

### Search results

Of the 120, 883 articles identified by the electronic database search, 27 met the inclusion criteria.

### Description of reviewed studies

Most studies were published between 2004 and December 2021 (*n* = 23) and were conducted in the WHO European Region (*n* = 11). The WHO Region of the Americas had the second highest number of studies (*n* = 8), with the majority conducted in the US (*n* = 7). Most studies employed a cross-sectional survey design (*n* = 9), followed by prospective cohort (*n* = 5), mixed method (*n* = 4), focus groups (*n* = 4), interviews (*n* = 3), and secondary data analysis (*n* = 2) designs. Measures of cigarette dependence were included in four studies and were gathered using the Fagerström Test for Nicotine Dependence. Motivation to quit was measured using the Stages of Change Model and was included in one study. The most studied population were nurses (*n =* 17), followed by doctors and nurses (*n =* 3), nursing students (*n =* 2), doctors (*n* = 2) and medical students (*n* = 1). Health professionals as a group comprising doctors, nurses, midwives, dentists, pharmacists, community healthcare workers, and allied health professionals were the subject of (*n =* 2) studies. Most studies included current smoking health professionals (*n* = 11), compared smoking health professionals and to non-smoking health professionals (*n* = 7), compared current and former smoking health professionals (*n* = 6), and one study compared nurses who smoke to smokers in the general population. One study compared former and never smoking health professionals, and one study included former smoking health professionals only. In the 19 studies that provided a definition for a smoker, criteria varied in terms of the number of cigarettes smoked, frequency, and recall periods. Eight studies did not include a definition of smoker.

The results from the quantitative studies are summarised in supplementary file 1, and the qualitative results are summarised in supplementary file 2.

### Predictors of cessation

A total of 13 quantitative studies examined a range of predictors related to health professionals’ own smoking cessation. The same 13 studies are included in the three sections below (predictors of intention to quit, quit attempts, and quit attempt success, see supplementary file 1).

#### Intention to quit

Four studies examined a range of demographic, attitude, and knowledge variables as predictors of intention to quit.

Only one included study examined age and gender as predictors and found no association with intention to quit [48].

Motivation to quit was examined in one study using the Stages of Change Model and found a positive association between nurses who smoked and were in the contemplative stage and those that would like to attend a smoking clinic [49].

One included study examined nicotine dependence as a predictor and found no association with desire to quit or motivation to quit after adjusting for age, gender, ethnicity, and medical speciality [50].

A single study examined quit attempts and found doctors who had more than five quit attempts had higher odds for desire to quit compared to doctors who had not tried to quit smoking [48].

Knowledge and concern about the harms of smoking were examined in two studies. One found smoking medical students in China who reported higher levels of knowledge about the hazards of smoking were more likely to indicate an intention to quit smoking [51]. Another found doctors who were concerned about the health harms associated with smoking had higher odds for intention to quit compared to doctors who were not concerned [48].

Having high levels of agreement with the responsibility to set a good example as a health professional was positively associated with intention to quit among doctors in Estonia [48], as was having a positive anti-smoking attitude for medical students [51].

#### Quit attempts

Four studies examined a range of demographic, current smoking, and knowledge variables as predictors of quit attempts.

Age and level of education were examined in one cohort study. Researchers found being an older smoker, but not level of education, was a factor related to attrition of participants between the first and second assessments of quit attempts [52].

The two studies that examined nicotine dependence gave inconsistent results. One found that being a nurse who smoked within 30 minutes of waking did not predict making a quit attempt in the previous 12 months but did predict making at least one quit attempt in their lifetime. Further, compared with women in the general population, nurses who had lower levels of nicotine dependence were less likely to have made a quit attempt in the previous 12 months [53]. In the same study, researchers found female nurses reported a significantly higher level of worksite support for quitting compared to females in the general population. Level of worksite support for quitting as a predictor was not directly examined to determine an association with quit attempts. By contrast, the other study on doctors in Estonia found no association between nicotine dependence and number of previous quit attempts [50].

A single study examined knowledge of smoking and smokeless tobacco form (i.e. chewing tobacco) to be harmful for COVID-19 and found a positive association in doctors and nurses in India [54].

#### Quit attempt success

Ten studies examined a range of demographic, attitude, and context variables as predictors of quit success.

The two studies that examined age found being an older smoker was predictive of a greater likelihood of having quit attempt success [52,55]. One study examined level of education as a predictor of quit attempt success and found no association [52].

The one study that examined attitude towards smoking bans found no association. Doctors and nurses who had a negative attitude towards the smoking ban were found to quit at an equivalent rate to those who had a more positive attitude toward the ban at baseline [52].

Only one included study examined attitudes towards cessation practice. This cohort study found nurses who believed that medical professionals should not smoke had a higher rate of attrition of participants between the first and second assessments of quit attempt success [56].

A single study examined prior quit attempts and found no association with quit attempt success [56].

The influence of the workplace having a smoke-free policy was examined in five studies, with inconsistent results. Two studies found a significant decrease in smoking prevalence among current smoking doctors and nurses in the US [52] and health professionals in Vietnam [57] in response to the smoking ban in hospital sites. Two found an association with decreased tobacco use during work hours but not overall prevalence. For example, Ryan et al. found changing habits in response to tobacco restrictive worksite policies was the only factor associated with decreased tobacco use among military hospital healthcare professionals [58]. The authors defined changing habits as changing the timing, location, and type of tobacco used. Indeed, in that study the predictor least identified as a motivator to quit was the tobacco restrictive worksite policies. Similarly, a study conducted in France found the smoke-free law was associated with decreased tobacco consumption during work hours, but not smoking prevalence among nurses who smoke [59]. One study in the UK found the reduction in work-time cigarette consumption was not statistically significant among nurses [60].

There was some evidence that work factors and the workplace environment were associated with quit attempt success. Three prospective cohort studies examined hours worked per week, night shift frequency, heavy physical work, work demands, role conflict, level of control, social support climate, exposure to threats and violence, commitment to work unit, and mastery of work as predictors for smoking cessation among nurses and nurses’ aides. One found that compared with working less than 10 hours per week (1–9), working 19–36 hours per week and working more than 36 of hours per week were associated with reduced odds of smoking cessation, after adjusting for daily consumption of cigarettes at baseline, age, gender, marital status, and having preschool children [61]. The other study found exposure to threats and violence at work, a perceived lack of a supportive, trustful, and relaxed work unit was associated with increased risk of smoking relapse after adjusting for age, gender, marital status, and having preschool children [62]. A cohort study conducted in Denmark found the factors related to current smoking nurses successfully quitting included low prior tobacco consumption at baseline, working day shifts, self-reported minimal physical job strain, and perceiving having some or much influence on their own work [55].

### Processes informing attitudes and beliefs

No studies were found that directly examined the processes informing the attitudes and personal and normative beliefs of smoking health professionals. All the quantitative studies were cross-sectional making it difficult to ascertain the causal links for smoking health professionals’ cessation. However, mixed method and qualitative studies did provide some insights (see supplementary file 2).

Six studies identified nurses’ internalised feelings of guilt and shame about their smoking in terms of the impact on their own health [63–65], and their failure to quit smoking despite being a nurse [66–68]. In one study, a current smoking nurse expressed their anticipatory shame if they were to be caught smoking by peers and friends who know them as a non-smoker, indicating they only smoke at home when there was no one around [64]. Similarly, current and former smoking nurses who expressed guilt and shame for their smoking described the pressure and frustration from friends and family expecting nurses ought to ‘know better than to smoke’ (p.391) [67]. Nurses who smoke also reported a perceived lack of understanding by managers and non-smoking colleagues about the support required for smoking cessation [67–71].

Four qualitative studies identified nurses’ awareness of the increased social unacceptability of smoking as a key factor influencing their decision about quitting. Some current smoking nurses indicated interest for a cessation program designed by and for nurses, although they expressed concern about it being public knowledge that nurses still smoke [67]. Indeed, former smoking nurses and nursing students noted social factors (e.g. the growing number of non-smokers in society, increasing cost of cigarettes, and smoke-free laws in bars and restaurants) as more important influences on their cessation attempts compared with nurses and nursing students currently smoking [67,72]. Currently smoking nurses noted they were aware of the reduced social acceptability of smoking, but that this was not a strong enough factor to get them to quit smoking [73]. In fact, some registered nurses and nursing students who smoke noted the social pressure to quit paradoxically produced a greater desire to keep smoking [74].

## Discussion

To our knowledge, this is the first review to synthesise the predictors and mechanisms from a psychological perspective that may impact smoking cessation in health professionals. Our findings suggest that limited quantitative research provides some indication of the factors that may influence smoking health professionals’ cessation. Other qualitative research included in this review provides insights into the attitudes and beliefs of smoking health professionals. For example, there was some evidence that being older predicted the likelihood of quit attempt success, although this was examined in only two studies. Additionally, having a smoke-free policy and an awareness of the increased social unacceptability of smoking did not predict quit attempt success consistently. Further, this review also found evidence that health professionals’ work environment predicted likelihood of quit attempt success. However, the research falls short of generating findings that can inform and guide the development of behavioural change interventions taking into account the struggle between their own personal smoking behaviour which is at odds with their expected health promotion roles. Prevalence estimates and individual predictors of smoking behaviour have remained the primary focus of smoking health professional research. Very few of the articles investigated the relative influence of group and other contextual factors. Further, most research was cross-sectional, indicating that much remains to be examined regarding the mechanisms and causal links for smoking health professionals’ cessation. Despite the limitations of the research to date, the thematic synthesis of the literature highlighted important insights and allows for recommendations for future research.

Very few studies examined the predictors of cessation of smoking by health professionals, including specifically the predictors of quit attempts and quit attempt success. Identifying predictors of quit success is important to improve interventions [36] and to assist matching people who smoke with approaches more likely to help them quit [75]. Given the limited number of studies, it is not possible to make definitive conclusions about the individual predictors that have been examined to date, nor to make a useful comparison with the factors predicting quit attempts and quit attempt success in general population samples [35,36,76]. Certainly, prospective research is necessary to establish which individual predictors are most likely to be associated with successful quit attempts in health professionals. For example, a review by Vangeli and colleagues found that a consistent predictor for quit success in general population samples is lower levels of tobacco dependence [36]. It remains unclear as to whether this would generalise specifically to health professionals. Moreover, socio-demographic variables (i.e. gender, age, marital status, and education levels) are not consistently associated with quit attempts or quit success in the general public across countries. It should also be noted that greater confidence in quitting was predictive of making a quit attempt in East Asian general population samples but not in westernised countries. Prospective research with respect to health professionals ought to include culture, both as a potential moderator, and the source of culture-specific variables that could explain quit success in respective societies.

Further, given some evidence for the potential impact of workplace climate on quit success identified in this review, organisational predictors for quit attempts and quit attempt success also warrant further inquiry; and these studies too ought to include culture, in view of the relationship of culture with job characteristics and workplace conditions on well-being for nurses [77,78]. In addition, the significance of culture and context in terms of health profession occupation groups should also be considered. Health professional occupation groups differ in how cessation responsibilities and roles are divided across different healthcare systems globally. For example, in some countries, some nurses are able to work independently with patients, while in others, nurses are limited in their positions.

This review found some evidence of tobacco smoking stigma experiences among nurses and nursing students who smoke, including, uniquely negative consequences given the role they occupy within the healthcare systems. Qualitative research conducted with current and former smoking nurses reported them experiencing feelings of guilt, shame, and hypocrisy about their smoking behaviour and their expected role. These experiences are consistent with the stigmatisation of smokers in both general population [33] and lung cancer patient samples [79,80]. Importantly, these experiences include both self and public stigma as potential help seeking barriers [33]. However, the extent to which this stigma, internalised as guilt, shame, and hypocrisy, actually influences smoking health professionals’ own cessation is unclear. Although some qualitative studies reported an influence among current and former smoking nurses, it is not possible on the basis of research to date to confirm or quantify an effect more generally or across other health professionals. This deficit in the research should be addressed urgently. In particular, the research lacks evidence regarding the effects of stigma on other health professional groups such as doctors, medical students, allied health, and other support staff.

Limitations in the research with regards to the definition of being a current smoker were noted. Specifically, there was a lack of standardisation across studies in the definition of a current smoker, which included variations in the number of cigarettes smoked, frequency, and recall periods. This methodological issue has also been noted in other reviews [27]. Relatedly, a substantial proportion of studies did not separate daily and occasional smokers to categorise current smokers, which is potentially problematic for how smoking health professionals self-identify and subsequent engagement with cessation support. There is evidence to suggest that there is a discrepancy between smoking identity and cigarette use in the general population, specifically for those who smoke occasionally [81]. This not only has implications for data collection but also for how interventions may be directed.

### Recommendations for future research

Based on the findings of this review, we propose a series of recommendations to health system managers and tobacco cessation researchers for future research. First, future research urgently needs to move towards an improved understanding and identification of the relevant factors associated with cessation in this population. Previous research has been almost universally atheoretical. More research is needed to identify the underlying processes and dynamics germane to health professionals who currently smoke or have previously smoked conventional and new forms of nicotine delivery (i.e. e-cigarettes and heated tobacco products). One direction for future research is to move towards qualitative research methods, such as grounded theory, to assist with theory building. Specifically, future research needs to examine more the beliefs espoused by health professionals about tobacco use, cessation, and their own roles as health professionals. Such beliefs about the nature of their roles within their professional networks seemed to predict both own cessation and provision of cessation guidance to others.

Next, future studies need to move beyond descriptive research that uses demographic characteristics as proxies of psychographic variables to predict smoking behaviour of smoking health professionals. Rather, the relevant predictors established through theory building should be systematically tested to determine quit success outcomes. In particular, future research needs to take a multi-focal perspective, examining the antecedents of attitudes and beliefs, the role of social networks, and factors at organisational, social, and cultural levels rather than individual-level factors alone. This could be achieved by testing multi-level models in prospective studies, informed by appropriate theoretical advances in attitudes and a wider range of predictors relevant to health professionals’ own cessation.

### Limitations

As with all research, this review has limitations. First, the focus of this review was to identify and assess the factors that may impact cessation for health professionals who smoke. A substantial proportion of publications were excluded from this review due to their focus of smoking prevalence only. Additionally, only articles published in English were included. Although a greater proportion of research was conducted in the WHO European Region, it is possible publications in low- and low- to middle-income countries (e.g. Asian and African populations) were overlooked. This is particularly important given the higher prevalence rates and limited funding for the types of tobacco control initiatives middle- to high-income countries can invest in and sustain [20]. Therefore, the role of health professionals providing cessation support is likely to be even more critical, as is an understanding of the relevant factors related to cessation in these cultures for health professionals who smoke. Lastly, this review did not analyse the differences in role and position of specific occupational groups of health professionals in various healthcare systems globally. Further studies are needed to examine the specific influence of culture and context in this regard.

### Conclusion

Although targeted cessation support for health professionals who smoke is desperately needed, this review suggests the evidence for factors predicting quit success is still limited. Despite the limited literature explicitly examining the relevant predictors of quit attempts and quit attempt success, being older and work environment factors were found to predict quit attempt success in some health professional groups. However, beyond those limited general findings, more research is urgently needed for improved understanding and identification of relevant factors. Theoretical work is required to identify the relevant predictors. Finally, more prospective research designs that take a multi-focal perspective are needed, examining the antecedents of currently and formerly smoking health professionals’ attitudes and beliefs, the role of social networks, and factors at organisational, social, and cultural levels rather than the individual level alone.

## Supplementary Material

Supplemental Material

## Paper context

Direct smoking cessation intervention support oriented on smoking health professionals is essential to ensure the optimal promotion of global cessation yet remains limited. This review aimed at synthesising evidence on the factors that predict smoking health professionals’ quit attempts and quit success to develop intervention support that respond to the unique needs of this population. Prospective research examining the relative influence of individual, group, and contextual variables is recommended to clarify the targets for change.

## Appendix A

Preferred Reporting Items for Systematic reviews and Meta-Analyses extension for Scoping Reviews (PRISMA-ScR) Checklist

**Table:**

SECTION	ITEM	PRISMA-ScR CHECKLIST ITEM	REPORTED ON PAGE #
**TITLE**			
Title	1	Identify the report as a scoping review.	1
**ABSTRACT**			
Structured summary	2	Provide a structured summary that includes (as applicable): background, objectives, eligibility criteria, sources of evidence, charting methods, results, and conclusions that relate to the review questions and objectives.	2
**INTRODUCTION**			
Rationale	3	Describe the rationale for the review in the context of what is already known. Explain why the review questions/objectives lend themselves to a scoping review approach.	4
Objectives	4	Provide an explicit statement of the questions and objectives being addressed with reference to their key elements (e.g. population or participants, concepts, and context) or other relevant key elements used to conceptualize the review questions and/or objectives.	5
**METHODS**			
Protocol and registration	5	Indicate whether a review protocol exists; state if and where it can be accessed (e.g. a Web address); and if available, provide registration information, including the registration number.	N/A
Eligibility criteria	6	Specify characteristics of the sources of evidence used as eligibility criteria (e.g. years considered, language, and publication status), and provide a rationale.	7
Information sources*	7	Describe all information sources in the search (e.g. databases with dates of coverage and contact with authors to identify additional sources), as well as the date the most recent search was executed.	6
Search	8	Present the full electronic search strategy for at least 1 database, including any limits used, such that it could be repeated.	Appendix B
Selection of sources of evidence^†^	9	State the process for selecting sources of evidence (i.e. screening and eligibility) included in the scoping review.	9
Data charting process^‡^	10	Describe the methods of charting data from the included sources of evidence (e.g. calibrated forms or forms that have been tested by the team before their use, and whether data charting was done independently or in duplicate) and any processes for obtaining and confirming data from investigators.	10
Data items	11	List and define all variables for which data were sought and any assumptions and simplifications made.	10
Critical appraisal of individual sources of evidence^§^	12	If done, provide a rationale for conducting a critical appraisal of included sources of evidence; describe the methods used and how this information was used in any data synthesis (if appropriate).	N/A
Synthesis of results	13	Describe the methods of handling and summarizing the data that were charted.	10
**RESULTS**			
Selection of sources of evidence	14	Give numbers of sources of evidence screened, assessed for eligibility, and included in the review, with reasons for exclusions at each stage, ideally using a flow diagram.	Figure 1
Characteristics of sources of evidence	15	For each source of evidence, present characteristics for which data were charted and provide the citations.	11
Critical appraisal within sources of evidence	16	If done, present data on critical appraisal of included sources of evidence (see item 12).	N/A
Results of individual sources of evidence	17	For each included source of evidence, present the relevant data that were charted that relate to the review questions and objectives.	Table S1 & S2
Synthesis of results	18	Summarize and/or present the charting results as they relate to the review questions and objectives.	12–17
**DISCUSSION**			
Summary of evidence	19	Summarize the main results (including an overview of concepts, themes, and types of evidence available), link to the review questions and objectives, and consider the relevance to key groups.	18
Limitations	20	Discuss the limitations of the scoping review process.	22
Conclusions	21	Provide a general interpretation of the results with respect to the review questions and objectives, as well as potential implications and/or next steps.	22
**FUNDING**			
Funding	22	Describe sources of funding for the included sources of evidence, as well as sources of funding for the scoping review. Describe the role of the funders of the scoping review.	24

JBI = Joanna Briggs Institute; PRISMA-ScR = Preferred Reporting Items for Systematic reviews and Meta-Analyses extension for Scoping Reviews.

*Where sources of evidence (see second footnote) are compiled from, such as bibliographic databases, social media platforms, and Web sites.

^†^A more inclusive/heterogeneous term used to account for the different types of evidence or data sources (e.g. quantitative and/or qualitative research, expert opinion, and policy documents) that may be eligible in a scoping review as opposed to only studies. This is not to be confused with information sources (see first footnote).

^‡^The frameworks by Arksey and O’Malley (6) and Levac and colleagues (7) and the JBI guidance (4, 5) refer to the process of data extraction in a scoping review as data charting.

^§^The process of systematically examining research evidence to assess its validity, results, and relevance before using it to inform a decision. This term is used for items 12 and 19 instead of ’risk of bias’ (which is more applicable to systematic reviews of interventions) to include and acknowledge the various sources of evidence that may be used in a scoping review (e.g. quantitative and/or qualitative research, expert opinion, and policy document).

## Appendix B

**Database Search Strategy for PsycINFO:**

1. (Nurse* or registered nurse* or primary care nurse* or nursing staff or nursing profession or physician* or primary care physician* or doctor* or healthcare worker* or healthcare professional* or health care professional* or health professional* or health service* or medical profession* or hospital* or hospital sites or teaching hospitals or smoke free hospitals or medical student*).ab. or (Nurse* or registered nurse* or primary care nurse* or nursing staff or nursing profession or physician* or primary care physician* or doctor* or healthcare worker* or healthcare professional* or health care professional* or health professional* or health service* or medical profession* or hospital* or hospital sites or teaching hospitals or smoke free hospitals or medical student*).ti. or (Nurse* or registered nurse* or primary care nurse* or nursing staff or nursing profession or physician* or primary care physician* or doctor* or healthcare worker* or healthcare professional* or health care professional* or health professional* or health service* or medical profession* or hospital* or hospital sites or teaching hospitals or smoke free hospitals or medical student*).id. (356375)
2. limit 1 to yr=‘1990 -Current’ (293695)
3. (Smok* or smoking behaviour or smoking behavior or smoking habits or tobacco or nicotine or cigarette or tobacco control or smoking cessation or tobacco denormalization or tobacco denormalisation or smoking denormalization or smoking denormalisation or moralisation of smoking or moralization of smoking).ab. or (Smok* or smoking behaviour or smoking behavior or smoking habits or tobacco or nicotine or cigarette or tobacco control or smoking cessation or tobacco denormalization or tobacco denormalisation or smoking denormalization or smoking denormalisation or moralisation of smoking or moralization of smoking).ti. or (Smok* or smoking behaviour or smoking behavior or smoking habits or tobacco or nicotine or cigarette or tobacco control or smoking cessation or tobacco denormalization or tobacco denormalisation or smoking denormalization or smoking denormalisation or moralisation of smoking or moralization of smoking).id. (67013)
4. limit 3 to yr=‘1990 -Current’ (61295)
5. 2 and 4 (6649)

This search was conducted on 17/06/19 and exported into EndNote
