# Supplementary material for: Factors associated with cessation of smoking in health professionals: a scoping review
Source: Glob Health Action. 2023 May 31;16(1):2216068. doi: 10.1080/16549716.2023.2216068 (PMC10234132; doi:10.1080/16549716.2023.2216068)
Supplement: Supplemental Material [file ZGHA_A_2216068_SM6656.zip › Supplementary_file_1_Table.docx]

Table 1. Results of quantitative studies on predictors of cessation in health professionals

| **Author/s**  **Year**  **Country**  **WHO Region** | **Year data gathered and sample** | **Smoking prevalence** | **Predictors** | **Outcome** | **Main findings** |
| --- | --- | --- | --- | --- | --- |
| An et al. (2015), Vietnam (WRP) | 2009 Survey:  *n* = 258 doctors;  *n* = 642 nurses and other health professionals not specified  2010 Survey:  *n* = 200 doctors;  *n* = 669 nurses and other health professionals not specified | 2009 Survey:  Currently smoking  (35.2% male; 1.1% female)  2010 Survey:  Currently smoking  (20.1% male; 0.2% female) | Smoke-free hospital | Successful cessation | Significant decrease in total prevalence of surveyed male and female health professionals who currently smoke (14.8% to 7.3%)  NS^d^ for number of cigarettes smoked per day among surveyed male health professionals (8.7 vs. 10.3)  NS^d^ for number of cigarettes smoked per day at work among surveyed health professionals (4.0 vs. 3.4) |
| Beletsioti‐Stika et al. (2006), Greece (EUR) | 2002  *n* = 308 nurses (clinical nurses, nurse managers and educators) | Nurses who currently smoke (46%); previously smoked (22%); have never smoked (31%) | Smoking status | Interest in  attending a  smoking  cessation clinic | *p* = 0.006^d^ positive association between currently smoking nurses in the contemplation stage and those who would like to attend a smoking cessation clinic |
| Eriksen (2005), Norway (EUR) | Base line 1999:  *n* = 2,720 nurses’ aides  Follow up 2001:  *n* = 2,275 nurses’ aides | Base line:  2,720 nurses’ aides who smoke daily  Follow up:  2,275 nurses’ aides who smoke daily | Working hours per week, age, gender, marital status, having preschool children, daily consumption of cigarettes | Successful cessation (Smoking zero cigarettes per  day) | *p* < 0.05^a^ Negative association with hours of work per week and the odds of smoking cessation in nurses’ aides. Compared with working 1-9 hours per week, working 10-36 hours per week, and working more than 36 hours per week were associated with reduced odds of smoking cessation, after adjusting for daily consumption of cigarettes at baseline, age, gender, marital status, and having preschool children |
| Eriksen (2006), Norway (EUR) | Base line 1999:  *n* = 1,373 nurses’  aides  Follow up 2001:  *n* = 1,203 nurses’  aides | Base line:  1,373 nurses’ aides who previously smoked  Follow up:  1,203 nurses’ aides who previously smoked | Age, gender, marital status, having preschool children, work demands, positive challenges in the job, role conflict, social climate in the work unit, exposure to threats and violence, fairness of immediate supervisor, supportive immediate supervisor | Occurrence of smoking relapse  (Smoking at least one cigarette a day at follow up) | p < 0.05^a^ Positive association with frequent exposure to threats and violence at work and the lowest quintile of the social climate index (supportive, trustful, relaxed) after adjusting for age, gender, marital status, and having preschool children |
| Fathallah et al. (2012), France (EUR) | 2008 Survey:  *n* = 454 nurses  2010 Survey:  *n* = 607 nurses | 2008 Survey:  Nurses who currently smoke (29.5%); previously smoked (24.9%); have never smoked (45.6%)  2010 Survey:  Nurses who currently smoke (30.1%); previously smoked (25.5%); have never smoked (44.3%) | Smoke-free hospital | Decreased smoking prevalence | NS^e^ decrease in smoking prevalence among nurses who currently smoke between 2008 and 2010, but a reduction in tobacco consumption during work hours and daily cigarette consumption |
| Kitajima et al. (2002), Japan (WPR) | Baseline 1997 and 1998:  *n* = 1,488 female nurses  Follow up 1999 and 2000:  *n* =1,195 female nurses | Baseline:  Nurses who currently smoke (32%); previously smoked (23%); have never smoked (45%)  Follow up:  Nurses who currently smoke (34%); previously smoked (27%); have never smoked (39%) | Nurses should not smoke as medical professional, females should not smoke, living with family | Successful cessation | *p* < 0.05^a^ Factors related to first survey nurses who smoke quitting before second survey, included nurses should not smoke as medical professionals, and living with family |
| Naik et al (2021), India (SEAR) | 2020  *n* = 116 doctors and nurses (modern medicine, nursing, dental, ayurveda, yoga & naturopathy, unani, siddha and homoeopathy) | Doctors and nurses who currently smoke (23.4%); smoke daily  (16.9%); have ever smoked (32.6%) | Age (<30), gender, occupation (doctor), marital status, vulnerable population at home, tobacco form, nicotine dependence, ever tried to quit tobacco, reduced tobacco use during COVID-19 pandemic, perceived tobacco as risk factor for COVID-19 cause/severity perceived tobacco form that increases | Reduced tobacco use during the COVID-19 pandemic  Cessation attempt during the COVID-19 pandemic | *p* < 0.05^b^ Positive association with presence of vulnerable population at home, ever quit attempt and ever diagnosed with COVID-19  *p* < 0.0^b^ Positive association with reduced tobacco use during the pandemic and perception both smoking and smokeless tobacco form increase the risk of COVID-19 |
|  |  |  | the risk for COVID-19, history of unprotected contact with COVID-19 case (yes), history if ever diagnosed with COVID-19 (yes) |  |  |
| Pold et al. (2018), Estonia (EUR) | 2002 Survey:  *n* = 322 doctors (family, specialist, dentist) who currently smoke  2014 Survey:  *n* = 189 doctors (family, specialist, dentist) who currently smoke | 2002 Survey:  Doctors who smoke occasionally (35.7%); daily (64.3%)  2014 Survey:  Doctors who smoke occasionally (22.2%); daily (77.8%) | Smoking frequency, gender, age, ethnicity, and place of residency, medical speciality, concern about harms of smoking, previous quit attempts, motivation to quit (personal health problems, marital, set a good example), study year (2002 or 2014) | Desire to quit | *p* < 0.05^a^ Positive association with doctors concerned about harms of smoking compared to doctors who were not concerned after adjusting for gender, age, and study year  *p* < 0.05^a^ Positive association with doctors who had 1-2, 3-4, and > 5 attempts to give up smoking compared to doctors who had not tried to quit smoking after adjusting for gender, age, and study year  *p* < 0.05^a^ Positive association with agreeing that setting a good example was a reason to quit smoking after adjusting for gender, age, and study year  After adjusting for all variables, daily smoking was not associated with higher desire to quit smoking but wish to set a good example was (*p* < 0.05^a^)  NS^a^ Gender, age, or ethnicity  ***Cessation Intention***  55.3% of smoking doctors reported a desire to quit smoking in 2002 and 52.9% in 2014 |
| Pold et al. (2020), | 2014 | 171 doctors who smoke daily | Desire to quit, number of previous | Nicotine dependence | NS^b^ with the desire to quit after adjusting for age, gender, ethnicity, medical speciality |
| Estonia (EUR) | *n =* 171 doctors (family, specialist, dentist) |  | quit attempts, motives to quit | (low and moderate) | NS^b^ with motives to quit after adjusting for age, gender, ethnicity, medical speciality  NS^b^ with number of previous quit attempts after adjusting for age, gender, ethnicity, medical speciality |
| Ryan et al. (2017), USA (AMR) | 2012  *n* = 2,074 military healthcare professionals | Military healthcare professionals who currently smoke (19.8%) | Resources used to support tobacco cessation (healthcare provider, counsellor, medication), factors reported as motivating tobacco cessation (health, fitness, children, family or friends, tobacco prices, tobacco policies), and self-reported changing habits | Decreased tobacco use in the past year | *p* < 0.05^b^ Positive association with self-reported changing habits (changing the timing or duration of work breaks, changing the location of tobacco use, or changing the type of tobacco used) in response to worksite policy  NS*** Resources used, motivations to quit tobacco use after adjusting for gender, age, and worker type |
| Sanderson et al. (2005), Denmark (EUR) | Baseline 1993:  *n* = 4,713 nurses who currently smoke  Follow up 1999:  *n* = 4,713 nurses | Baseline:  Nurses who currently smoke (36%)  Follow up:  Nurses who currently smoke (27%; of the baseline currently smoking sample, | Age, tobacco consumption per day, years of smoking, spouse/partner’s recent occupation, fresh fruit consumption, high blood pressure, shift work, physical job strain, personal | Successful cessation | *p* = 0.002^a^ Positive association with older age (57-66 years)  *p* < 0.001^a^ Positive association with low tobacco use (1-14 g/day) at baseline  *p* < 0.001^a^ Positive association with short duration of smoking  (> 20 years)  *p* = 0.016^a^ Positive association with partner was retired  *p* = 0.002^a^ Positive association with daily fresh fruit consumption  *p* = 0.032^a^ Positive association with high blood pressure |
|  |  | 24% had quit by follow up) | perceived influence on own work |  | *p* = 0.014^a^ Positive association with working day shifts  *p* = 0.014^a^ Positive association with having low physical job strain  *p* = 0.011^a^ Positive association with perceiving having some influence on own work |
| Sarna et al. (2012), USA (AMR) | 2006/2007  *n* = 2,566 female nurses (registered and licensed practical nurses)  *n* = 93,717 female general population (not nurses or other healthcare professionals) | Currently smoking female nurses (12.1%)  Currently smoking female general population (16.6%) | Nicotine dependence  (high – smoking within 30 minutes of waking up; low – smoking after 30 minutes of waking up) | Quit attempt:  Ever tried to  quit smoking  Quit attempt:  Tried to quit within the last 12 months | *p* < 0.0001^a^ Positive association for nurses with a high level of nicotine dependence compared to other females after controlling for demographics (age, race, marital status) and smoking characteristics (age of smoking initiation, smoking 12 or more days a month)  NS^a^ among either group with low level of nicotine dependence  *p* = 0.04^a^ Negative association for nurses with a low level of nicotine dependence compared to other females after controlling for demographics (region) and smoking characteristics (years of smoking, cigarettes per day, age of smoking initiation, smoking 12 or more days a month)  NS^a^ among either group with high level of nicotine dependence |
| Stillman et al. (1994), USA  (AMR) | Baseline 1988:  *n* = 818 doctors  *n* = 1,048 nurses  Follow up 1989:  *n* = 517 doctors  *n* = 554 nurses | Baseline 1988:  Doctors who currently smoke (4.6%); nurses who currently smoke (16.4%)  Follow up 1989:  Doctors who currently smoke (2.1%); nurses who | Age, gender, occupation (doctor, nurse), smoking status  Age, occupation (doctor, nurse), education level, | Overall quitting behaviour from pre-ban to post-ban  Long-term quitting behaviour from | *p* = 0.03^b^ Positive association with being a doctor  *p* < 0.01^b^ Positive association with older age  NS^b^ Education level  NS^b^ Baseline attitude towards smoking ban  *p* = 0.009^b^ Positive association with being a doctor  *p =* 0.005^b^ Positive association with older age  NS^b^ Education level |
|  |  | currently smoke (11.7%) | smoking related attitudes | pre-ban to post-ban | NS^b^ Baseline attitude towards smoking ban |
| Strobl & Latter (1998), UK  (EUR) | Date not specified  *n =* 33 nurses who currently and previously smoked | Nurses who currently smoke (*n* = 28); previously smoked  (*n* = 3) | Smoke-free hospital | Decreased tobacco use in the past 9 months | NS^d^ Reduction in cigarette consumption per shift  NS^d^ Reduction in cigarette consumption during remainder of day  NS^d^ Reduction in cigarette consumption during the whole working day |
| Yang et al (2020), China (WPR) | 2018    *n* = 7,169 male medical students | Medical students who currently smoke (12.4%) | Age, knowledge, anti-smoking attitude, smoking behaviours and attitude of best friend towards smoking, been advised to quit smoking | Intention to quit | *p* < 0.05^c^ Positive association with higher level of knowledge about the hazards of smoking among smoking medical students  *p* < 0.05^c^ Positive association with having a positive anti-smoking attitude among smoking medical students  *p* < 0.05^c^ Positive association with ever been advised by others to quit among smoking medical students  *p* < 0.05^c^ Negative association with best friends approved of smoking among smoking medical students |

*Note:* NS = Not statistically significantly different; ^a^ Multivariate Logistic Regression; ^b^ Multivariable Logistic Regression; ^c^ Multilevel analysis; ^d^ Chi-squared or Fisher’s exact test; ^e^ ANOVA; AFR = Africa Region; AMR = Region of Americas; SEAR = South-East Asia Region; EUR = European Region; EMR = Eastern Mediterranean Region; WPR = Western Pacific Region.
