# Supplementary material for: Factors associated with cessation of smoking in health professionals: a scoping review
Source: Glob Health Action. 2023 May 31;16(1):2216068. doi: 10.1080/16549716.2023.2216068 (PMC10234132; doi:10.1080/16549716.2023.2216068)
Supplement: Supplemental Material [file ZGHA_A_2216068_SM6656.zip › Supplementary_file_2_Table.docx]

Table 2. Results of qualitative studies on mechanisms and predictors on cessation of smoking in health professionals

| **Author/s**  **Year**  **Country**  **WHO Region** | **Year data gathered** | **Participants** | **Aim of interview** | **Main themes and findings** |
| --- | --- | --- | --- | --- |
| Bialous et al. (2004), USA (AMR) | 2002 | *N* = 59 nurses (registered and licensed practical nurses)  30 currently smoke and 29 have previously smoked | Issues related to nurses’ attitudes toward smoking, quitting, and preferences for smoking cessation interventions | *Myths and misconceptions about quitting*  Several nurses stated they lacked the knowledge and supporting resources, including colleagues’ support, for their quit attempts.  *Over-coming addiction*  Participants expressed shame and guilt about their smoking and described the pressure and frustration from friends and family who assumed nurses know better than to smoke. Former smoking nurses noted social forces, the growing number of non-smokers, an important influence for quitting, while current smoking nurses indicated this the least important influence.  *Strategies to enhance successful cessation*  Participants were enthusiastic about a cessation program by and for nurses, with some concern that public knowledge that nurses still smoke could be detrimental to the professional image. |
| Gebhardt et al (2014), USA (AMR) | 2008 | *N* = 12 nursing students  7 previously smoked and 5 have never smoked | Better understand nursing students’ knowledge about smoking behaviours and quitting | *Motivators to quit*  Former smoking participants noted motivators for quitting was their current nursing education, the cost of cigarettes, smoke-free policies and laws, and isolation from social situations. Participants also noted feeling shame and a reduced credibility to their employer, co-workers, patients, and family members if they were observed smoking.  *Barriers to quit*  Many indicated the association between going out to drink alcohol and smoking cigarettes a key barrier.  *Strategies used to quit*  Participants noted refraining from social activities involving alcohol, nicotine replacement therapy, alternative medicine strategies, and supportive relationships. |
| Gifford et al. (2013), New Zealand (WPR) | 2012 | *N* = 201 nurses (registered and enrolled), student nurses, mid-wives, and community healthcare workers  43 currently smoking, 32 have never smoked, and 126 previously smoked | The context of smoking for Maori nurses and the impact of cessation interventions, national regulation, and workplace policies on quitting | *Personal stories of quitting*  Currently smoking participants described how they continued to smoke despite knowing the health risks and having a desire to quit. Some participants also described potential embarrassment should they be caught smoking by peers and friends who know them as a non-smoker, choosing to only smoke at home when no-one is around.  *Dissatisfaction with current quit strategies*  Some participants rejected current quit strategies as too judgemental, punitive, and stigmatising.  *Future cessation strategies*  Participants noted the importance of addressing underlying reasons for smoking, i.e., the social determinants and appropriate support. |
| González et al. (2009), Spain (EUR) | 2007 | *N* = 15 female nurses (primary healthcare)  All currently smoking | Perception  of nurses who are smokers concerning the suitability of their anti-smoking therapeutic relationships with their clients, and factors of cessation | *Providing cessation support as a smoker – help or hinderance*  Many participants indicated a reluctance to provide cessation support to patients due to experiencing guilt for being a smoker themselves. Other participants felt their smoking assisted in therapeutic relationship as they can empathise with the patient. Some noted they need to be able to differentiate their personal from professional life.  *Cessation training*  A considerable number indicated they avoided cessation training information as it made them feel uncomfortable. Almost none of the participants reported that they lacked information about the harmful effects of smoking.  *Cessation contradiction*  Participants described chastising patients smoking in the street, who in turn pointed out their hypocrisy. Participants minimised their own smoking with the fact that they are not the ones in hospital. |
| Harrison et al. (1991), Canada (AMR) | 1990 | *N* = 84 nurses (registered) and nursing students  All currently smoking | Smoking prevalence and other smoking behaviour among Canadian nurses | *Own cessation*  The majority indicated they want to quit. More nursing students than nurses tended to seek help to quit smoking. Several barriers to quitting were identified: (a) Public’s lack of understanding about the strength of smoking addiction, (b) Social pressure to quit paradoxically produced a greater desire to smoke, and (c) Quitting was associated with a major lifestyle change and changing social groups. |
| Heath et al. (2004), USA (AMR) | Date not specified | *N* = 12 nurses (nurse practitioners)  All currently smoking | How tobacco-dependent nurses describe their own experiences of tobacco dependence and cessation | *Living as an insider in the world of addiction*  Although financial and health consequences were discussed, the most prevailing cost concerned relationships with others, family, friends, professional relationships, and associates. Some noted, despite their knowledge of how destructive smoking is, they continue to smoke, and is an aspect of their life they cannot control.  *Having the outside-in view of living with tobacco addiction*  Some noted people they meet at work assume they do not smoke because they do not look like a smoker. All nurses discussed how supportive and positive comments made by family and colleagues made more of an impact when attempting to quit.  *Being caught in the middle of tobacco addiction*  All participants described an awareness of the paradox of being a tobacco-dependent healthcare provider. Some described a time “in the good old days” where smoking was acceptable in the hospital for patients and nurses. All participants indicated not wanting patients to know they smoke to avoid the impression it is ok to smoke because they do. |
| Mark et al. (2005), Australia (WPR) | 2000-2002 | *N* = 4 female Aboriginal health workers  All currently smoking | Barriers facing Aboriginal Health Workers who want to quit smoking | *Barriers to quitting*  Stress as well as lack of support from family members or work colleagues was mentioned as barriers to quitting.  *Quit attempts*  All participants had tried to quit smoking without support. Nicotine Replacement Therapy was mentioned by one participant but indicated it is costly and is not effective. |
| Mujika et al. (2017), Spain (EUR) | 2010 | *N* = 11 nurses  6 currently smoking and 5 previously smoked | Psychosocial component around nurses’ smoking behaviour and their health promotion role | *Being “one of them” and professional identity*  Some separated their personal and professional identities, isolating their smoking behaviour from their profession. Many expressed concerns that patients may notice their smoking status which inhibited their ability to engage in cessation intervention with patients due to hypocrisy.  *Duality in psychological effect of smoking*  The act of smoking had both a perceived positive effect, an escape, and negative feelings of guilt and shame. The negative feeling was partly related to concern about the impact on their personal health and the image of nursing they may be reflecting. |
| Petersen et al. (2020), Multiple (EUR) | 2015-2016 | *N* = 82 nurses  53 currently smoking and 29 previously smoked | Perceptions of workplace factors that influence quitting behaviours | *Effect of smoking on patient interactions*  Participants described feelings of guilt, embarrassment, and shame in response to patient’s reactions to the smell of cigarette smoke on their person.  *Perceived collegial support for quitting*  Many participants reported intentionally avoiding informing colleagues they were quitting, and when aware, support from co-workers were not helpful. Some expressed having low expectations of the workplace supporting their quit attempts.  *Impact of work policies*  Participants reported the workplace could be associated with the maintenance of smoking behaviours, including high levels of stress, low professional status, nursing shortages. While some reported smoking restriction policies to be supportive of quitting, some reported feeling devalued and discriminated against. |
| Rezk-Hanna et al. (2018), Multiple  (EUR) | 2015-2016 | *N* = 81 nurses  53 currently smoking and 29 previously smoked | Attitudes, barriers, and facilitators to quit efforts of current and former smoking nurses | *Barriers to nurses quitting*  Many acknowledged their motivation to continue was to remain part to a community of nurses who smoke at work. Lack of support in the home environment emerged as a strong barrier. |
|  |  |  |  | *Facilitators to nurses quitting*  Former smoking participants acknowledged quitting was hard, but a key motivator was concern for their own personal health. Support and encouragement, particularly at home was another motivating factor to quit. They described feeling shame when their children asked why they smoked, or when they smoked in their presence. Pregnancy was another motivating factor. |
| Sarna et al. (2005), USA (AMR) | 2002 | *N* = 60 nurses (registered and licensed practical nurses)  30 currently smoking and 30 previously smoked | Attitudes and experiences of nurses regarding smoking and smoking cessation in the workplace | *Stress and smoking*  One participant indicated stress was a barrier for a nurse to quit. Former smoking participants acknowledge quitting was the most difficult thing to do especially in the face of work stress, and that the workplace is a place for relapse.  *Support from colleagues and quitting*  Participants described lack of support from colleagues, especially when undergoing withdrawal. The loss of relationships was another identified barrier.    *Hiding from patients and their families*  Current and former smoking participants described guilt and shame about their smoking and the strategies they used to hide their smoking, especially from patients and families.  *Impact of workplace smoking restrictions*  Participants who smoke were ambivalent about smoking restrictions in the hospital. Numerous participants described a lack of trust in institutional support for cessation efforts.  *Challenges to cessation in the workplace*  Participants noted the 12-hour work shifts and lack of resources in the workplace as barriers to cessation. Some viewed administration involvement in smoking cessation as a deprivation of rights. On the other hand, several mentioned the importance of administration and colleagues’ understanding of the difficulty in quitting and success of cessation efforts. |
| Veny et al. (2011), Spain (EUR) | 2007 | *N* = 15 nurses (primary health care)  All currently smoking | Views of nurses on factors moderating smoking cessation | *Willingness to quit smoking*  Almost all the participants who smoke were thinking of quitting sooner or later. A key basis for success according to the nurses was a firm decision of wanting to quit. Almost all indicated they had tried to quit smoking in their lifetime.  *Reasons to quit smoking*  Fear of health deterioration was a common reason for giving up smoking, however, most  noted this was not enough to quit smoking, and they would not quit until the health problem manifested. There was a fear of lung cancer. Most quit smoking or significantly decreased their smoking when they became pregnant, describing they did not want to harm the child.  *Health professionals as a healthy model*  Participants indicated smoking in public is frowned upon lately and has worse connotations if the smoker is a nurse. The shame of their own smoking and providing cessation advice to patients was a factor for quitting.    *Contradictions, excuses, and self-justification*  Many participants did not feel discriminated by the colleagues for smoking, however, indicated they tried not to smoke at work. Some indicated they know that smoking is harmful and that they should quit but that it did not help being constantly reprimanded. Some described their experience with relapse, feeling ashamed on relapsing more so because they are a nurse. |

*Note:* AFR = Africa Region; AMR = Region of Americas; SEAR = South-East Asia Region; EUR = European Region; EMR = Eastern Mediterranean Region; WPR = Western Pacific Region.
